# Digital rights and mobile health in Southeast Asia: A scoping review

**DOI:** 10.1177/20552076241257058

**Published:** 2024-05-28

**Authors:** Adam Poulsen, Yun JC Song, Eduard Fosch-Villaronga, Haley M LaMonica, Olivia Iannelli, Mafruha Alam, Ian B Hickie

**Affiliations:** 1 Brain and Mind Centre, 4334 The University of Sydney, Sydney, Australia; 2 eLaw Center for Law and Digital Technologies, 4496 Leiden University, Leiden, Netherlands

**Keywords:** Human rights, mHealth, scoping review, LMICs, Southeast Asia, data privacy

## Abstract

**Objective:**

Digital technology has the potential to support or infringe upon human rights. The ubiquity of mobile technology in low- and middle-income countries (LMICs) presents an opportunity to leverage mobile health (mHealth) interventions to reach remote populations and enable them to exercise human rights. Yet, simultaneously, the proliferation of mHealth results in expanding sensitive datasets and data processing, which risks endangering rights. The promotion of digital health often centers on its role in enhancing rights and health equity, particularly in LMICs. However, the interplay between mHealth in LMICs and digital rights is underexplored. The objective of this scoping review is to bridge this gap and identify digital rights topics in the 2022 literature on mHealth in Southeast Asian LMICs. Furthermore, it aims to highlight the importance of patient empowerment and data protection in mHealth and related policies in LMICs.

**Methods:**

This review follows Arksey and O’Malley's framework for scoping reviews. Search results are reported using the PRISMA-ScR (Preferred Reporting Items for Systematic Reviews and Meta-Analyses Extension for Scoping Reviews) checklist. Frequency and content analyses were applied to summarize and interpret the data.

**Results:**

Three key findings emerge from this review. First, the digital rights topics covered in the literature are sparse, sporadic, and unsystematic. Second, despite significant concerns surrounding data privacy in Southeast Asian LMICs, no article in this review explores challenges to data privacy. Third, all included articles state or allude to the role of mHealth in advancing the right to health.

**Conclusions:**

Engagement in digital rights topics in the literature on mHealth in Southeast Asian mHealth is limited and irregular. Researchers and practitioners lack guidance, collective understanding, and shared language to proactively examine and communicate digital rights topics in mHealth in LMIC research. A systematic method for engaging with digital rights in this context is required going forward.

## Introduction

In 2012, the United Nations officially recognized digital rights, in the resolution on ‘The Promotion, Protection and Enjoyment of Human Rights on the Internet’.^1^ The resolution affirmed that the internet and other technologies are important tools for exercising human rights and that “the same rights that people have offline must also be protected online”.^1^ Following this landmark resolution, the United Nations further affirmed the importance of digital rights protections and called for state adoption in 2016,^2^ and later reiterated that digital technologies have the potential to both advance and violate human rights in the 2020 ‘Road Map for Digital Cooperation’.^3,4^ In 2022, the European Commission codified digital rights by signing ‘The Declaration on Digital Rights and Principles for the Digital Decade’.^5^ Crucially, the declaration supports the principles established by the United Nations and reshapes and applies existing human rights to the digital space. Notably, it extends the right to privacy to the right to the protection of personal data online, and it broadens the right to freedom of expression to the right to freedom of expression in the online environment.^5^ Digital rights can be divided into two categories. First, existing human rights which are extended to, realized in, and protected in digital environments. Second, emerging human rights to access, use, and participate in digital environments (e.g., the right to internet access).^6^ For the purposes of this study, digital rights are “all existing and emerging human rights that extend to, and relate to accessing and protecting, participation in the digital space”.^7^

The emerging right to internet access pervades much of the discussion about digital rights.^8–10^ Although several countries have recognized the right to internet access including Greece^11^ and India,^12^ the status of the right to internet access on the international level is still being debated.^13^ Neither the United Nations nor the European Commission explicitly proclaims and codifies ‘the right to internet access,’ but both call for necessary regulatory protections of internet access to preserve or at least not deliberately inhibit human rights.^3,5,14^ Internet access as a right is a well-explored topic in the literature, especially in research addressing low- and middle-income countries (LMICs) which experience a high risk of regular digital rights violations due to restrictive policies that impose, for instance, internet shutdowns and online content censorship.^15–17^ It is typical for ‘online’ to be conflated with ‘digital’ in discussions about digital rights, leading to the common perception that digital rights are exclusively centered on the internet.^18^ However, digital rights also concern data justice, communication rights, labor rights, privacy, democratization issues, and so on.^19^ Other information and communications technology ecosystems and related digital rights considerations include, for instance, healthcare systems and respect for human dignity^20^; surveillance technologies and freedom of movement^21^; and automation and the right to work.^22^ The indistinctness between the concepts of ‘online’ and ‘digital’ relating to rights is particularly noticeable in the academic literature.^18^

Acknowledging that the discussion about digital rights is often centered around the ‘Western world’ and that rights are perceived and defined differently in other regions,^23^ this work seeks to create an understanding of digital rights topics within the context of Southeast Asia. According to a four-item classification for digital rights presented in an article addressing digital rights advocacy in Southeast Asia, digital rights are the following: (1) conventional rights translated to digital spaces, (2) data-centered rights (e.g., data security and privacy), (3) rights to access digital spaces and services and meaningful participation, and (4) rights to participate in the governance of the digital or the internet.^18^ At present, only the first group—conventional rights translated to digital spaces—is at the center of the digital rights movement in Southeast Asia, and a lack of technical capacity (i.e., knowledge and skills relating to technology) is a major gap in addressing digital rights violations in the region.^18^

In 2023, the United Nations released the ‘Human Rights Impacts of New Technologies on Civic Space in Southeast Asia’ report, presenting six trends affecting human rights online in the region.^24^ Those trends are: (1) the spread of hateful, misogynistic, and discriminatory content, (2) organized and coordinated online attacks and harassment, (3) technologies of surveillance, (4), restrictive legal and regulatory frameworks, (5) criminalization and prosecution of online expression, and (6) internet shutdowns and network interference. The report draws attention to the role of both state and non-state actors in using digital tools to undermine democratic governance and highlights recent policies and regulations governing online spaces which, in some cases, reinforce current restrictions of freedoms of expression, association and privacy. Another article noted this extension of “imposing draconian laws” into online governance policy in Southeast Asia.^18^ For example, libel laws have become cyber libel laws, which may discourage online dissenting voices in, for instance, Brunei, Malaysia, and the Philippines.^25,26^

According to the ‘Freedom on the Net 2022’ report, which measures each country's level of internet freedom based on limits on content, violations of user rights, and obstacles to access, no Southeast Asian country assessed scores high enough to be considered ‘free.’ Most score as ‘partly free’ (Cambodia, Indonesia, Philippines, Malaysia, Singapore), some score as ‘not free’ (Myanmar, Thailand, Vietnam), and others are not assessed (Brunei, Timor-Leste, Laos).^27^ Key internet controls employed by the majority of Southeast Asian governments that negatively affect internet freedom include content blocking to restrict access to political, social, or religious information; technical attacks against government critics or human rights organizations; online discussion manipulation by pro-government commentators; and arrest, imprisonment, prolonged detention, physical attack, or killing of bloggers or information and communications technology users for sharing political or social content.^27^

The ubiquity of mobile technology and mobile health (mHealth) interventions in LMICs, presents an opportunity to reach remote populations and enable them to exercise civil and political rights and economic, social, and cultural rights.^28,29^ Digital health, such as mHealth, is frequently presented as a tool for advancing human rights and health equity,^30^ as well as supporting fairness, ecological sustainability, and local autonomy.^31^ Research has demonstrated the benefits of mHealth to disease management,^32,33^ including improving medication adherence,^34^ health education,^35^ and access to personal health information and health services through various interoperable technologies, such as wearable sensors^36^ and cloud computing.^37^ Yet, concurrently, the all-pervasiveness of mobile phones and rapid implementation of mHealth entails processing large amounts of sensitive data which can threaten rights, including the right to privacy and non-discrimination, as the growing volume of this data becomes increasingly vulnerable to breaches of privacy and information security.^38^ Further, despite the popularity of mobile technology, devices are not created to be inclusive of the economic, social, and cultural rights and civil and political rights of vulnerable groups, such as older adults living in LMICs.^39^ In the face of salient digital rights concerns in LMICs, the rights of digital health users are inadequately regulated in LMICs.^40,41^ Exemplary general digital rights concerns affecting mHealth in this context include mandatory Subscriber Identity Module (SIM) card registration; gender disparities and the gender digital divide; online surveillance; cyberviolence conducted via mobile phones; online content censorship; prolonged data retention; and mobile app download taxes.^42–44^ As an example, mandatory SIM card registration negatively impacts the right to privacy (having to identify oneself for connectivity), the right to the protection of personal data online (having to entrust personal data to mobile carriers), and freedom of expression in the online environment (concerns about surveillance over mobile communications and internet).^42^ In the Southeast Asian context, mandatory SIM card registration and the potential subsequent impacts on mHealth access and digital rights are particularly salient given that all eleven countries in the region have implemented this policy as of 2022.^45^

In the research space, knowledge about the engagement with digital rights topics in the literature on mHealth in LMICs is currently underexplored. This scoping review aims to address this gap by reviewing the 2022 literature on mHealth in LMICs and identifying digital rights topics. To limit the scope of the review to digital rights topics exclusively, regularly discussed mobile access topics (e.g., cost, connectivity, infrastructure, and literacy) are not examined. Additionally, only literature published in 2022 is included due to resource limitations and the high interest in the subject of digital rights at present. Lastly, the scope is narrowed to only include selected articles from Southeast Asia.

In the Methods section of this article, the study method is presented. Then, the findings are reported in the Results section. Next, in Discussion section, the findings are discussed and future research directions are considered. Finally, the Conclusion section concludes this article with a summary of the key study findings and implications.

## Methods

This study adheres to the framework and recommendations for scoping reviews outlined by Arksey and OMalley^5^ and Levac, Colquhoun, and O’Brien.^47^ As such, this section reports the following six methodological stages: (1) identifying the research question; (2) identifying relevant studies; (3) study selection; (4) charting the data; (5) collating, summarizing, and reporting results; and (6) consultation. Several recommendations by Levac, Colquhoun, and O’Brien^47^ were adopted, including explicitly articulating the research question, having two authors independently review full-text articles for inclusion, and piloting data extraction with five included articles and then reviewing the data chartering form to ensure it is consistent with the research question and purpose. The full description of the mixed methods employed in this study is presented in the study protocol for the larger scoping review published elsewhere.^7^ Focusing on Southeast Asia, nine selected articles from Southeast Asia were identified and included here from the larger review.

### Identifying the research question

The guiding question of the review is ‘What digital rights topics have been explored in the 2022 literature on mHealth in LMICs?’ The research question was developed in discussion with the research team upon reflection on particular policies in some LMICs that risk restricting digital rights and thus access to mHealth, such as mobile app download taxes and mandatory SIM card registration. Southeast Asia was chosen as a focus here due to the significant digital rights concerns and substantial mHealth implementation in the region.

### Identifying relevant studies

Searches were performed on 25 November 2022, 9 March 2023, and 16 August 2023, throughout seven electronic databases: Web of Science, Scopus, Ovid, ACM Digital Library, IEEE Xplore, ProQuest, and PubMed. The search query included terms that described the subjects of interest and the nature of the research question. It included numerous abbreviations and variations (e.g., “LMIC,” “low- and middle-income countr*,” “low-income countr*,” and “developing country”) and was adapted to the requirements of each database. Search results were collated and imported into Covidence, a web-based tool for joint article screening, reviewing, and data collection. Automated and manual removal of citation duplicates was conducted on Covidence. Article screening was performed in a two-stage process by two reviewers (AP and YJCS) to evaluate the relevance of the articles to the research question. Title and abstract screening was conducted first, followed by full-text article screening. The reviewers regularly met to resolve any emerging disagreements related to article selection. An arbiter (EFV) resolved any disagreements that could not be settled by the reviewers in those meetings.

### Study selection

The following inclusion criteria were applied: (1) the article was peer-reviewed; (2) the article was published in the English language; (3) the article was published in 2022; and (4) the article addressed the subjects of interest (i.e., mHealth in LMICs and digital rights topics). Articles were excluded if they did not address mHealth in LMICs (e.g., reviews on health technology in LMICs not covering mHealth) or digital rights topics specifically (e.g., mobile phone accessibility topics such as cost and digital literacy).

### Charting the data

For included articles, data extraction was independently piloted with five articles by the two reviewers to reach a consensus and check consistency. Afterwards, one reviewer (AP) completed the data extraction of the remaining articles. Three co-authors (AP, YJCS, and HML) developed a data chartering form, determining the data items to be collected during data extraction.

### Collating, summarizing, and reporting results

All data were exported and collated in Microsoft Excel for frequency and content analysis. Descriptive statistics were calculated to summarize the general characteristics of the included articles. Content analysis was applied to interpret the extracted data. Regular meetings with the research team were held to support data analysis and interpretation.

### Consultation

No consultation with stakeholders was conducted at any stage during this scoping review.

## Results

### Search and selection of literature

**Figure 1.**
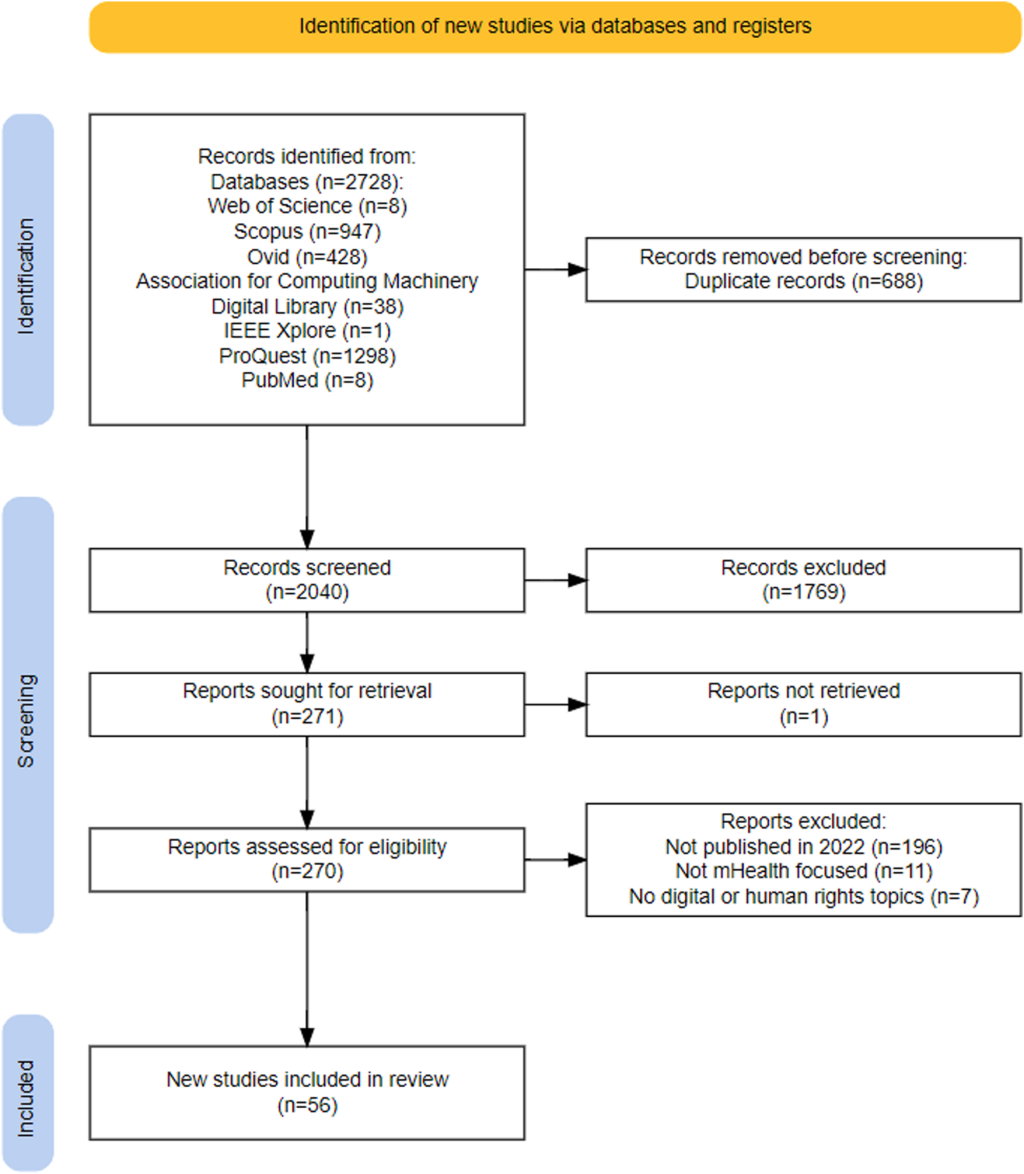
PRISMA-ScR flow diagram of the scoping review process. Generated by authors using the Shiny app for producing PRISMA 2020-compliant flow diagrams.^48^

Searches of seven databases yielded 2728 articles. After removing duplicates, 2040 articles were screened by title and abstract. This resulted in 271 articles being eligible for full-text article screening, although only 270 were retrieved as one article was inaccessible and thus excluded. Before full-text screening, post hoc eligibility criteria were applied to narrow the review to 2022 publications only, excluding 196 articles. The remaining 74 articles were screened by full text, which resulted in 56 articles being included in the review. Of those 56 articles, nine selected articles from Southeast Asia were identified and included here. Figure 1 reports the scoping review process using the Preferred Reporting Items for Systematic Reviews and Meta-Analyses Extension for Scoping Reviews (PRISMA-ScR) flow diagram.

### General characteristics of included articles

Table 1 describes the general characteristics of the included articles. All articles included in this review were journal articles published in 2022. Included articles reported varied study designs, with multiple following randomized controlled trials (*n*  =  2), qualitative research (*n*  =  2), and systematic reviews (*n*  =  2). Based on the first author's affiliation country, the articles originated from various countries, with multiple coming from Indonesia (*n*  =  2), the United States (*n*  =  2), and Australia (*n*  =  2).

**Table 1.** General characteristics of the included articles.

Article characteristics		Number of articles out of *N* = 9
Publication type	Journal article	9 (100%)
Study design	Randomized controlled trial	2 (22%)
	Qualitative research	2 (22%)
	Systematic review	2 (22%)
	Sequential explanatory mixed method	1 (11%)
	Observational	1 (11%)
	Scoping review	1 (11%)
First author's affiliation country	Indonesia	2 (22%)
	United States	2 (22%)
	Australia	2 (22%)
	Germany	1 (11%)
	Malaysia	1 (11%)
	Singapore	1 (11%)

Table 2 reports the Southeast Asian LMICs implicated in the included articles. A total of five countries were implicated in the articles. Indonesia was considered the most, being examined in four articles. Cambodia (*n*  =  3) and Malaysia (*n*  =  2) were also mentioned in multiple articles. The remaining countries, Thailand and Vietnam, were implicated in only one article. One review article was assigned multiple characteristics as it addressed Thailand, Indonesia, and Malaysia.

**Table 2.** Southeast Asian LMICs implicated in included articles.

Article characteristics		Number of articles out of *N* = 9
Southeast Asian LMICs implicated	Indonesia^49–52^	4 (44%)
	Cambodia^53–55^	3 (33%)
	Malaysia^52,56^	2 (22%)
	Vietnam^57^	1 (11%)
	Thailand^52^	1 (11%)

### mHealth characteristics

Table 3 reports the mHealth characteristics of the included articles. Characteristics include (1) the health area addressed through mHealth, (2) mHealth technology, (3) mHealth functions, (4) mHealth purpose/application, and (5) target mHealth end users. In several cases, articles were assigned multiple characteristics.

**Table 3.** Mhealth characteristics of included articles.

Article characteristics		Number of articles out of *N* = 9
Health area	Community health	5 (55%)
	Health education	5 (55%)
	Health promotion	4 (44%)
	Maternal and child health	4 (44%)
	Sexual and reproductive health	3 (33%)
	Global health	2 (22%)
	Gender-based violence health	2 (22%)
	Health policy	1 (11%)
	Mental health	1 (11%)
	Physical health	1 (11%)
mHealth technology	Smartphone	7 (77%)
	Featurephone	3 (33%)
	Unspecific mobile phone	2 (22%)
	Tablet	1 (11%)
	Wearable technology	1 (11%)
	Telephone	1 (11%)
mHealth functions	Smartphone app-based	7 (77%)
	Short Message Service (SMS)	5 (55%)
	Voice communication	4 (44%)
	Audio clips	3 (33%)
	Interactive electronic client lists	2 (22%)
	Interactive Voice Response (IVR)	2 (22%)
	Multimedia Messaging Service (MMS)	1 (11%)
	Tethered accessory sensors	1 (11%)
	Built-in accelerometer	1 (11%)
	Mobile money transfers and banking services	1 (11%)
mHealth purpose/application	Client education and behavior change	6 (66%)
	Provider-to-client communication	5 (55%)
	Data collection and reporting	4 (44%)
	Electronic health records	2 (22%)
	Information seeking	2 (22%)
	Electronic decision support	2 (22%)
	Sensors and point-of-care diagnostics	1 (11%)
	Provider-to-provider communication	1 (11%)
	Provider work planning and scheduling	1 (11%)
	Human resource management	1 (11%)
	Client-to-client communication	1 (11%)
Target mHealth end users	General public	7 (77%)
	Community health worker	3 (33%)
	Healthcare service client/patient	1 (11%)
	Allied health professional	1 (11%)

On health areas, the majority of articles aimed to address community health (*n*  =  5) and health education (n  =  5) with mHealth. The next most salient health areas covered were health promotion (*n*  =  4) and maternal and child health (*n*  =  4). Sexual and reproductive health (*n*  =  3), global health (*n*  =  2), and gender-based violence health (*n*  =  2) were also mentioned in multiple articles. Health policy, mental health, and physical health only emerged in one article each.

On mHealth technology, most articles discussed smartphones (*n*  =  7). Featurephones were mentioned in several articles (*n*  =  3), as well as unspecific mobile phones (*n*  =  2). Tablets, wearable technologies, and telephones were cited in one article each.

On mHealth functions, most articles discussed smartphone app-based functions (*n*  =  7). Multiple articles mentioned Short Message Service (SMS, including alerts and reminders) (*n*  =  5), voice communication (*n*  =  4), audio clips (*n*  =  3), interactive electronic client lists (*n*  =  2), and Interactive Voice Response (IVR) (*n*  =  2). The following functions were examined in one article each: Multimedia Messaging Service (MMS), tethered accessory sensors (including wearable technology and implantable devices), built-in accelerometers, and mobile money transfers and banking services.

On mHealth purpose/application, client education and behavior change (including treatment adherence) were mentioned in most articles (*n*  =  6). The next most salient mHealth purpose/application explored was provider-to-client communication (online doctor consultation, digital prescription delivery, etc.) (*n*  =  5), followed by data collection and reporting (*n*  =  4). Several articles cited electronic health records (*n*  =  2), information seeking (*n*  =  2), and electronic decision support (information, protocols, algorithms, checklists, etc.) (*n*  =  2). Sensors and point-of-care diagnostics, provider-to-provider communication (user groups, consultation, etc.), provider work planning and scheduling, human resource management, and client-to-client communication (e.g., enabling connection of groups of clients) were discussed in one article each.

On target mHealth end users, most articles discussed the general public as target users (*n*  =  7). Multiple articles cited community health workers (*n*  =  3). The following target users were mentioned in one article each: healthcare service clients/patients and allied health professionals (dietitians, physiotherapists, podiatrists, etc.).

### Digital rights characteristics

Table 4 describes the digital rights characteristics of the included articles. Characteristics include (1) terms used, (2) explicit digital rights topics cited, and (3) implied digital rights topics. The language used to categorize the rights topics is informed by the broader human and digital rights literature, policy, and advocacy, including documentation from the Association of Southeast Asian Nations Civil Society Conference/Peoples’ Forum,^58^ Association for Progressive Communications,^59^ European Commission,^5^ and EngageMedia.^60^ In several cases, articles were assigned multiple rights characteristics as several explicit and implied digital rights topics emerged in individual articles.

**Table 4.** Digital rights characteristics of included articles.

Article characteristics		Number of articles out of *N* = 9
Terms used	None	8 (88%)
	Human right	1 (11%)
	Digital right	0 (0%)
	Digital human right	0 (0%)
Explicit digital rights topics cited	Empowerment	3 (33%)
	Right to health	1 (11%)
	Right to privacy	1 (11%)
	Freedom of choice	1 (11%)
	Digital gender divide	1 (11%)
	Freedom of movement	1 (11%)
Implied digital rights topics	Right to health	9 (100%)
	Solidarity and inclusion	6 (66%)
	A fair digital environment	3 (33%)
	Right to social security	3 (33%)
	Digital education, training and skills	2 (22%)
	Freedom from discrimination	2 (22%)
	A protected, safe and secure digital environment	2 (22%)
	Freedom of choice	2 (22%)
	Right to privacy	1 (11%)
	Right to safe and healthy working conditions	1 (11%)
	Right to internet access	1 (11%)

#### Terms used

None of the articles explicitly used the terms ‘digital right’ or ‘digital human right,’ and only one article used the term ‘human right.’ To follow is the only instance ‘human right’ was explicitly used, extracted from an Indonesian study:

“Another health problem in Indonesia is nutritional problems, including stunting and wasting. These health problems are important because health is one of the human rights and has an essential contribution to the development and progress of a nation”.^49^

#### Explicit digital rights topics cited

On explicit digital rights topics cited, *empowerment* was the only topic explicitly cited in multiple articles (*n*  =  3). The following topics were explicitly cited in one article each: *right to health, right to privacy, freedom of choice, digital gender divide,* and *freedom of movement*.

Empowerment was referenced and conceptualized in multiple ways: community empowerment, health patient empowerment, and women's empowerment. One study presents a smartphone app (iPosyandu) as a tool to support a community-based program that seeks to provide basic healthcare services and enable “community empowerment in health promotion strategies”.^49^ The authors link mHealth and empowerment, stating that mandatory smartphone training provided during participation in the program “conveys the spirit of community empowerment” through cooperation between cadres.^49^ Health patient empowerment through mHealth was referenced in another study, which explores the need for a smartphone app in stroke management for informal caregivers in Malaysia.^56^ The authors note that the use of digital health, particularly mHealth, creates opportunities for “empowering patients in terms of self-management and caregivers’ involvement, thus improving patients’ wellbeing”.^56^ In a systematic review of the role of mHealth in changing gender relations, the authors remark that increased access to mHealth “enhanced women's empowerment to make informed decisions about health care”.^57^ In a discussion about advancing women's self-efficacy with mHealth, one study reviewed by the authors centered on an SMS messaging-based maternal and child health program in Vietnam. The authors highlight that the Vietnam study led to women users reporting feeling “empowered by this newfound knowledge, made informed decisions about health care, and were more confident in their interaction with community health workers”.^57^

One study explicitly mentioned the right to health and linked it to mHealth.^49^ Namely, the authors suggest that mHealth is a tool to realize the right to health by complementing existing health services and improving health data collection, storage, and processing.

The right to privacy and the role of mHealth in ensuring privacy were mentioned in a systematic review of healthcare professional perspectives on mHealth-driven sexual and reproductive health (SRH) services in rural settings in LMICs, one of which was Cambodia.^55^ The authors reported that compared to in-person health consultations mHealth services advance privacy, confidentiality, and trust. Although a Cambodia study was included in the review, the authors did not relate the privacy considerations to that study. However, the review did cite freedom of choice and link it to the Cambodia study reviewed. The authors stated that SRH generally implies that people “have the capability to reproduce and the freedom to decide if, when, and how often to do so” and that mHealth specifically helps clients in making those health decisions, supporting freedom of choice.^55^

The digital gender divide and freedom of movement were cited in a systematic review, which included a Southeast Asian (Vietnam) study.^57^ The authors did not directly link these topics to Southeast Asia, rather the topics were cited when reporting on other studies or as generalizations applying to all included articles. The digital gender divide was cited using several different terms and multiple contexts connected to mHealth. This included the “gendered divide” related to mobile phone ownership affecting mHealth program enrolment, the reduction of the “gendered digital divide” due to gains in women's self-confidence and skills through mobile phone use and communication as a part of mHealth programs, the reinforcement of “existing gender-based inequities such as the digital divide” in mHealth initiatives that fail to overcome gender-based barriers, and the “gender-based digital divide” impacting mHealth generally.^57^ In generalizing all the included articles in the review, freedom of movement was referenced. The authors state that mHealth can reduce gender-based barriers and cite the lack of freedom of movement as one of those barriers.^57^ That review also referred to freedom of expression, however, it did not relate to the digital space. That is, the authors only cited freedom of expression when commenting broadly on the research study design, warning that conducting dyadic interviews with partnered participants risks one partner dominating the discussion and limiting the truthful responses of the other.^57^ As this topic was not linked to the digital space in the article, it is not included in the data.

#### Implied digital rights topics

On implied digital rights topics, the *right to health* was implied in all articles. The next most salient implied topic emerging in most articles was *solidarity and inclusion* (*n*  =  6). *A fair digital environment* and the *right to social security* were inferred from multiple articles (*n*  =  3), as well as ‘*digital education, training and skills,’ freedom from discrimination, ‘a protected, safe and secure digital environment,’* and *freedom of choice* across several articles (*n*  =  2). The following topics were implied in one article each: *right to privacy, right to safe and healthy working conditions,* and *right to internet access*. Table 5 reports the implied digital rights topics in the included articles, with an exemplary article extract and rationale for how each extract implies the associated topic.

## Discussion

This review aimed to address the gap in knowledge about the engagement with digital rights topics in the literature on mHealth in LMICs by reviewing articles published in 2022 relating to Southeast Asia and identifying digital rights topics. Three key findings emerge from this review. First, the digital rights topics covered in the literature on mHealth in Southeast Asian LMICs are sparse, sporadic, and unsystematic. Second, despite the saliency of data privacy concerns in Southeast Asian LMICs, no article explores challenges to data privacy. Third, either explicitly or implicitly, every article references the role of mHealth in advancing the right to health, in alignment with the existing literature emphasizing the urgency and intended role of mHealth in this context.

**Table 5.** Implied digital rights topics in included articles, with exemplary extract and rationale.

Implied digital rights topics	Article extracts	Rationale
Right to health	“[mHealth] apps have been widely used because they can collect data from end-users and store them in a source database…Data quality is a prerequisite for data analysis in medical research, preventing errors, and providing information for evidence-based intervention in health promotion”^50^	Links mHealth-based data collection to improved health data quality, and ultimately advancing health policy, provision, and intervention
Solidarity and inclusion	“mHealth is yet to address key public health challenges related to geographical and socioeconomic inequalities in access to preventive health”^52^	Reflects on mHealth excluding those experiencing socioeconomic inequalities
A fair digital environment	“By partnering with Indonesia's major telecommunication companies and GoI [Government], the sender of the SMS was always shown as ‘PKH [Program Keluarga Harapan] information’ in order to create trust in the reliability of the information source”^51^	Emphasizes the importance of data reliability to create the trustworthiness of mHealth-delivered information
Right to social security	“[There is] a lack of information and knowledge about how to apply for welfare aid. Caregivers were thus concerned with developing a stroke mobile application to meet this purpose”^56^	Considers a mHealth app providing welfare aid information
Digital education, training and skills	“Technological literacy is a skill needed to access digital technology, which is necessary for mHealth uptake”^55^	Notes that one factor impacting mHealth uptake depends on digital skills
Freedom from discrimination	“Health interventions using mobile phones…present a viable solution for connecting hard-to-reach, stigmatized, and criminalized populations”^53^	Explores how mHealth can support access to health services without discrimination
A protected, safe and secure digital environment	“In Cambodia, WhatsApp was selected [to support a mHealth intervention] because it is a secure end-to-end encrypted messaging service, which means only the two parties involved can see and read what is sent”^54^	Notes security measures to protect digitally- mediated sensitive information
Freedom of choice	“mHealth interventions have the potential to increase women's autonomy in seeking health services and health information, thus enhancing their health-related decision-making”^57^	Links mHealth to increasing autonomy and improved decision-making
Right to privacy	“[For a mHealth intervention] having a text and voice options may allow callers to have more privacy and disclose more personal information than a voice-only option”^54^	Suggests that text-based mHealth interventions protect one's privacy
Right to safe and healthy working conditions	“The pressure to drink alcohol at work from supervisors and peers is common for FEWs [female entertainment workers] in Cambodia…Messages developed…and sent out through the Mobile Link platform advised about subtly avoiding and reducing the effect of heavy drinking”^53^	Discusses the role of a WhatsApp chatline in delivering health information to improve safety and health at work
Right to internet access	“The availability of sophisticated and frequently updated technologies would aid in making [health] knowledge and information conveniently accessible over the internet for each individual in any situation, at any time, and everywhere…This is especially true for stroke victims and their caregivers in the context of healthcare”^56^	Considers that health literacy can be improved by greater internet access

This review highlights the scarcity of research explicitly using the terms ‘digital right,’ ‘human right,’ or ‘digital human right’ in this context (1/9 included articles). Multiple factors may contribute to the lack of engagement with these terms. First, the recognition, interpretation, and prioritization of human rights are influenced by cultural, social, political, and linguistic differences.^19^ As a result, human rights, and therefore digital rights, are often associated with “Western” individualistic ethics in many LMICs, including those in Southeast Asia.^61^ Second, the definition of digital rights and their relationship to human rights remains unsettled in Southeast Asia.^18^ Third, in the research space in LMICs, there is a lack of emphasis on digital rights due to a lack of digital rights policy and research funding and attention.^18^ Last, beyond the right to health, mHealth research does not often focus on human rights generally^62^ and thus researchers may not be compelled to engage with rights and related terms in an accountable, transparent, and direct way such as, for instance, health policymakers and providers should while following human rights-based approaches to health.^63^

As evidenced by this review, few digital rights topics are explicitly referenced and explored in this space. Specifically, the included articles explicitly cite empowerment the most (3/9 included articles), and to a lesser extent, remark on the right to health (1/9), the right to privacy (1/9), freedom of choice (1/9), digital gender divide (1/9), and freedom of movement (1/9). To a greater extent, various digital rights topics are implicated in the included articles, yet without being explicitly cited. The most implied digital rights topic was the right to health, which was implied in all included articles (9/9). After that, the topic of solidarity and inclusion was implied in most included articles (6/9). Multiple implicated ‘a fair digital environment’ (3/9), the right to social security (3/9), ‘digital education, training and skills’ (2/9), freedom from discrimination (2/9), ‘a protected, safe and secure digital environment’ (2/9), and freedom of choice (2/9). To a smaller extent, the right to privacy (1/9), the right to safe and healthy working conditions (1/9), and the right to internet access (1/9) were implied.

Failing to recognize and account for digital rights topics in mHealth in LMICs research risks missing opportunities to leverage mHealth to realize digital rights and risks exacerbating existing digital rights inequities. This is particularly concerning in the Southeast Asian LMIC context where digital rights are at significant risk.^18^ For example, the review shows limited engagement with the digital gender divide. Yet, research shows that there exists a mobile phone ownership gender disparity in several Southeast Asian LMICs, such as Indonesia where there is an 11% difference in mobile phone ownership, favoring men.^64^ Failing to acknowledge the digital gender divide and account for it in mHealth research risks excluding women without a mobile phone, thus creating a barrier to realizing the right to health and other rights via mHealth. Since Indonesia was at the center of nearly half of the included articles in this review (4/9) and none of those articles addressed the digital gender divide, the findings of this review suggest that Indonesian mHealth research is at risk of exacerbating gender disparities in this space. No specific policies regulate mHealth in Indonesia,^65,66^ and until such policies are developed to establish standards and guidance for mHealth research and implementation which accounts for the digital gender divide in the country this risk remains salient.

None of the articles included in this review explore challenges to data privacy. Yet, significant challenges and concerns surrounding data privacy persist in Southeast Asian LMICs affecting mHealth. Research addressing core digital rights considerations for advocacy in Southeast Asia designates data-centered rights—i.e., data privacy and security—as one of the four categories critical to conceptualizing digital rights in the region.^18^ Furthermore, as the United Nations points out, threats to data privacy are significant in Southeast Asia, with negative trends affecting digital rights including the use of technology for surveillance and criminalization and prosecution of online expression, restricting data privacy.^24^ Much like a lack of data privacy protection affects freedom of expression in, for instance, Brunei, Malaysia, and the Philippines,^25,26^ insufficient regulation around data privacy may impact mHealth users in the region. For example, those living with HIV in Vietnam, who fear stigmatization due to a lack of privacy at in-person clinics and prefer to use mHealth solutions for greater privacy,^41^ remain at risk of their data privacy being jeopardized due to poor cybersecurity policy and the absence of mHealth legislation in the country.^67^ Given the ongoing challenges to data privacy affecting mHealth in Southeast Asian LMICs, the lack of engagement with these challenges in the included articles is of concern.

All the included articles noted the role of mHealth in advancing the right to health in Southeast Asian LMICs, either explicitly (1/9 included articles) or implicitly (9/9). This trend aligns with the existing literature emphasizing the urgency and intended role of mHealth in this context.^28,29^ Noting the core components of the right to health outlined by the United Nations Committee on Economic, Social and Cultural Rights^68^ in the ‘The Right to the Highest Attainable Standard of Health’—health availability, accessibility, acceptability, and quality—the included articles broadly present mHealth as playing a role in realizing these core components. Specifically, the included articles linked the use of mHealth to improved availability of community health (5/9), health education (5/9), health promotion (4/9), maternal and child health (4/9), sexual and reproductive health (3/9), etc. mHealth users are provided greater accessibility via, for instance, smartphones (7/9) or featurephones (3/9), through the facilitation of quality, research-backed services, including client education and behavior change (6/9), provider-to-client communication (5/9), data collection and reporting (4/9), electronic health records (2/9), information seeking (2/9), etc. Primarily, the beneficiaries of mHealth in Southeast Asian LMICs are expected to be the general public (7/9) and community health workers (3/9), improving accessibility generally. Yet, continued failure to adequately engage with other aforementioned digital rights topics puts accessibility at risk. As the United Nations^68^ illustrates, health accessibility consists of four dimensions, i.e., non-discrimination, physical accessibility, economic accessibility (affordability), and information. As such, minimal engagement with freedom from discrimination in the included articles (2/9) risks impacting the role of mHealth in advancing the right to health in Southeast Asian LMICs.

Going forward, it is necessary to develop and adopt research methods and standards for reporting in mHealth research to systematically account for and accommodate digital rights. Otherwise, continued indifference to digital rights in mHealth research addressing Southeast Asian LMICs, as well as frequent digital rights violations and lack of policy to protect digital rights in these nations, put patient empowerment, data protection, and inclusion in mHealth interventions at risk. At the higher level, advancing policy, education, information and communications technology accessibility, and underlying infrastructure is essential to enabling digital rights in Southeast Asian LMICs. New policy to regulate digital rights as it relates to mHealth should be developed at both the regional and state levels, ensuring a harmonious, transparent, and rights-based approach to mHealth governance, design, development, and implementation throughout the region. Furthermore, it is vital to advance the definition of digital rights and their relationship to human rights in the region. In doing so, Southeast Asian LMICs have the opportunity to be proactive to digital rights in developing, for example, policy and research methods, rather than being reactive like most of the world. Additionally, Southeast Asian LMICs could be the first to adequately and methodically anticipate and consider marginalized communities in the digital rights space, such as Indigenous communities who are otherwise excluded, as evidenced in this review in which none of the included articles engaged with the codified rights of Indigenous peoples.

Several future research opportunities emerge from this study, which may expand on or enhance the work presented here. These include (1) a larger systematic review to build upon this scoping review; (2) an in-depth exploration of digital rights terminology, definitions, and relationship to human rights generally in Southeast Asia; (3) the development and evaluation of research methods and standards intended to systematically account for and support digital rights in mHealth research; and (4) an in-depth review and analysis of each country's mHealth research, practices, and policies.

### Strengths and limitations

This review has some strengths and limitations. It adhered to Arksey and O’Malley's framework for scoping reviews^46^ and adopted later recommendations by Levac, Colquhoun, and O'Brien.^47^ Seven multidisciplinary databases were searched on three instances across a 10-month period. Article screening and the data extraction pilot were performed by two independent reviewers to advance reliability. A diverse, multidisciplinary research team supported data analysis and interpretation during regular meetings throughout the duration of the study. Several limitations are noted. First, the inclusion of literature published in 2022 limits the generalizability of the study findings. Second, given that the selected articles from Southeast Asia included here are part of a larger review without regional limitations, the search strategy did not include terms to specifically target the Southeast Asian region or specific countries. This potentially excludes relevant articles not captured by the broad search strategy. Third, the review only included English-language, peer-reviewed literature, omitting other potentially relevant sources. Finally, unlike the systematic review method, the scoping review method does not include an assessment of the quality of the literature.^69^

## Conclusions

The recent literature on mHealth in Southeast Asian LMICs lacks systematic engagement with digital rights topics. This highlights a lack of focus and rights-based approaches in mHealth research in this context despite the saliency of digital rights concerns in Southeast Asian LMICs. None of the included articles explicitly referred to digital rights, and few explicitly cited and explored digital rights topics. Furthermore, none of the articles explored challenges to data privacy, despite the saliency of the issue in the region. However, all the articles situate mHealth in the role of advancing the right to health, either explicitly or implicitly. A systemic and proactive method for engaging with, and accounting for, digital rights in mHealth in LMICs research is needed to ensure an intentional, anticipatory, and shared understanding of digital rights and related impacts going forward.
